# Quantification of *Leishmania* (*Viannia*) Kinetoplast DNA in Ulcers of Cutaneous Leishmaniasis Reveals Inter-site and Inter-sampling Variability in Parasite Load

**DOI:** 10.1371/journal.pntd.0003936

**Published:** 2015-07-23

**Authors:** Milagros Suárez, Braulio M. Valencia, Marlene Jara, Milena Alba, Andrea K. Boggild, Jean-Claude Dujardin, Alejandro Llanos-Cuentas, Jorge Arevalo, Vanessa Adaui

**Affiliations:** 1 Instituto de Medicina Tropical Alexander von Humboldt, Universidad Peruana Cayetano Heredia, Lima, Peru; 2 Public Health Ontario Laboratories, Public Health Ontario, Toronto, Canada; 3 Department of Medicine, University of Toronto, Toronto, Canada; 4 Tropical Disease Unit, University Health Network, Toronto General Hospital, Toronto, Canada; 5 Department of Biomedical Sciences, Institute of Tropical Medicine, Antwerp, Belgium; 6 Department of Biomedical Sciences, University of Antwerp, Antwerp, Belgium; 7 Departamento de Ciencias Celulares y Moleculares, Facultad de Ciencias y Filosofía, Universidad Peruana Cayetano Heredia, Lima, Peru; US Food and Drug Administration, UNITED STATES

## Abstract

**Background:**

Cutaneous leishmaniasis (CL) is a skin disease caused by the protozoan parasite *Leishmania*. Few studies have assessed the influence of the sample collection site within the ulcer and the sampling method on the sensitivity of parasitological and molecular diagnostic techniques for CL. Sensitivity of the technique can be dependent upon the load and distribution of *Leishmania* amastigotes in the lesion.

**Methodology/Principal Findings:**

We applied a quantitative real-time PCR (qPCR) assay for *Leishmania* (*Viannia*) minicircle kinetoplast DNA (kDNA) detection and parasite load quantification in biopsy and scraping samples obtained from 3 sites within each ulcer (border, base, and center) as well as in cytology brush specimens taken from the ulcer base and center. A total of 248 lesion samples from 31 patients with laboratory confirmed CL of recent onset (≤3 months) were evaluated. The kDNA-qPCR detected *Leishmania* DNA in 97.6% (242/248) of the examined samples. Median parasite loads were significantly higher in the ulcer base and center than in the border in biopsies (*P*<0.0001) and scrapings (*P* = 0.0002). There was no significant difference in parasite load between the ulcer base and center (*P* = 0.80, 0.43, and 0.07 for biopsy, scraping, and cytology brush specimens, respectively). The parasite load varied significantly by sampling method: in the ulcer base and center, the descending order for the parasite load levels in samples was: cytology brushes, scrapings, and biopsies (*P*<0.0001); in the ulcer border, scrapings had higher parasite load than biopsies (*P*<0.0001). There was no difference in parasite load according to *L*. *braziliensis* and *L*. *peruviana* infections (*P* = 0.4).

**Conclusion/Significance:**

Our results suggest an uneven distribution of *Leishmania* amastigotes in acute CL ulcers, with higher parasite loads in the ulcer base and center, which has implications for bedside collection of diagnostic specimens. The use of scrapings and cytology brushes is recommended instead of the more invasive biopsy.

## Author Summary

Cutaneous leishmaniasis (CL) is a parasitic disease of the skin caused by obligate intra-macrophage protozoa of the genus *Leishmania* which usually presents as ulcerative lesions at the site of infection. Traditionally, histopathological and diagnostic studies on CL have employed samples collected from the border of the ulcer since this area is believed to contain the highest amount of parasites. However, no formal demonstration of the distribution of *Leishmania* parasites in the ulcer has been provided yet. Focusing on human skin lesions of recent onset (≤3 months) caused by *L*. (*Viannia*) species, we estimated the parasite loads among different skin lesion sites by means of quantitative real-time PCR targeting the parasite kinetoplast DNA. Paired lesion samples collected by use of different sampling methods were analyzed. We found that the ulcerated zone of the lesion contained a higher parasite load than the ulcer border, and that scraping and cytology brush specimens presented higher parasite loads as compared to the more invasive biopsy. Our results have implications for bedside collection of diagnostic and post-therapeutic follow-up specimens from CL patients.

## Introduction

Cutaneous leishmaniasis (CL) is a parasitic disease of significant public health problem in at least 18 countries of Latin America; about 67,000 CL cases were reported to occur annually in the last decade [1]. The disease is caused by protozoan parasites of the subgenera *Leishmania* (*Viannia*) and *L*. (*Leishmania*), with the former being responsible for most cases. The clinical phenotypes of CL are diverse and range from a single or few cutaneous ulcerative lesions at the site of infection that may heal spontaneously, diffuse and disseminated CL with multiple non-ulcerative lesions, to disfiguring mucocutaneous leishmaniasis that can be life-threatening [2,3]. The severity and outcome of the disease are dependent among others on the immune responses evoked by the host and the infecting *Leishmania* species [4,5].

Parasitological diagnosis of CL relies on either the microscopic demonstration of *Leishmania* amastigotes in cutaneous tissue or the isolation of parasites from lesions in culture [6–8]. While these techniques are highly specific for diagnosing CL, they are insufficiently sensitive [9]. Polymerase chain reaction (PCR)-based testing of skin lesion specimens has become an important tool to diagnose CL, because of its high sensitivity and specificity (up to 100%) [10–12]. Significant progress has been made towards evaluating molecular-based non-invasive methods for the diagnosis of CL that overcome the disadvantages of the traditional, invasive sampling methods such as punch biopsies, aspirates or skin slits/scrapings [13–16]. One such non-invasive method, cytology brush PCR, has shown potential for widespread use, both in the clinic [15] and field settings [17].

Several studies indicate that the sensitivity of diagnostic methods for CL can be dependent upon the number and dispersion of parasites in the lesion, the method used to sample ulcers, the stage (chronicity) of the lesion, and the technical skills of the personnel [6,9,10,11,18,19]. Conventionally, in accordance to guidelines established by the World Health Organization (WHO) [20], tissue samples have been obtained from the lesion border, where parasite load and the density of inflammatory mononuclear cells harboring parasites are thought to be higher [21]. Evidences supporting that other sampling sites within lesions could result in comparable or even increased sensitivity of parasite detection by microscopy or PCR have been provided [22,23]. In a recent study using quantitative real-time PCR (qPCR) targeting *Leishmania* 18S rDNA, it has been reported that swab sampling over the ulcer allowed to recover a higher amount of parasite DNA as compared to aspirate samples taken from the lesion border [16]. Whether this indicates differences inherent to the sampling methods or truly reflects a higher parasite load in the ulcerated zone of the lesion [16] needs to be ascertained.

The analysis of the load and distribution of *Leishmania* parasites within the skin lesions would be important not only for determining the best location within the ulcer to obtain samples for diagnostic purposes, but also for an eventual follow up of a patient’s response to treatment [24,25]. We herein applied a standardized qPCR assay targeting minicircle kinetoplast DNA (kDNA) [26] to detect and quantify *Leishmania* (*Viannia*) parasites in 3 sites within the CL ulcer (raised border, base, and center). Paired lesion samples were collected by use of different sampling methods: a punch biopsy and a dermal scraping from each of the 3 lesion sites, and a cytology brush from each the base and center of the ulcer. The parasite load levels were compared according to the ulcer site, sampling method, and the infecting *Leishmania* species. We restricted this study to lesions originated from patients with acute CL (≤3 months), which characteristically have high parasite load in contrast to lesions from patients with chronic disease (>6 months) [10,26]. This fact enabled detection of *Leishmania* and quantifiable parasite load levels in most clinical specimens. Importantly, early diagnosis is considered a desirable control measure for CL. To our knowledge, this is the first report that quantitatively compares the parasite loads among different skin lesion sites and sampling methods by means of qPCR, thereby providing an insight into the likely distribution of *Leishmania* amastigotes in the ulcer for the *L*. (*Viannia*) species present in our sample set. The implications of our results on diagnosis of CL and the prognostic applicability are discussed, as well as how they may relate to the immunopathology of the disease.

## Methods

### Ethics statement

This study was conducted according to the principles specified in the Declaration of Helsinki and under local ethical guidelines (Universidad Peruana Cayetano Heredia Institutional Review Board). The study protocol, informed consent and sampling procedures were approved by the Institutional Review Boards of the Hospital Nacional Cayetano Heredia and Universidad Peruana Cayetano Heredia (Lima, Peru) for studies involving human subjects. Written informed consent was obtained from all participants prior to enrolment.

### Sample size calculation

The sample size was calculated using the G*Power 3.1 software (release 3.1.9.2; available from: http://www.gpower.hhu.de/) [27] to assess the null hypothesis of no difference in parasite load levels between different sampling sites within the CL ulcer. Assuming a medium effect size of 0.5, a significance level of 5%, and a power of 80%, 35 matched pairs of lesion samples were required to be examined (two-sided, Wilcoxon signed-rank test for matched pairs). For significant results, the effect size was assumed to be ‘medium’, which means an effect visible to the naked eye. Non-significant results were assumed to have a ‘small’ effect size. We managed to study 31 paired lesion samples from patients presenting with acute CL.

### Patients

Patients that attended the Leishmaniasis Clinic at the Instituto de Medicina Tropical Alexander von Humboldt, Hospital Nacional Cayetano Heredia, in Lima, Peru, between January and June 2013 for the examination of skin lesions were invited to participate in the study and evaluated for possible eligibility. Patients were considered for enrolment if they presented with ulcerative skin lesions of recent onset (≤3 months of evolution), with elevated and infiltrative borders and a lesion size over 1 cm in diameter; and were able to give written informed consent for the sampling procedures. We included adult patients with laboratory confirmed diagnosis of CL, as defined by a positive result on at least 1 of these 3 tests: direct microscopy on Giemsa-stained lesion smears [7], lesion aspirate microculture [28], and qualitative PCR targeting kDNA minicircles [29] on a biopsy specimen obtained from the ulcer border. This diagnostic PCR includes internal control primers for amplifying the human beta-globin gene as previously described [13]. The intradermal leishmanin skin test (LST), used to assess exposure to *Leishmania* infection, was performed on CL patients before treatment, as described elsewhere [30,31]. We excluded patients allergic to local anesthetics, with clinical evidence of bacterial or fungal superinfection of the ulcer (when possible), with any contraindication to skin biopsy and those undergoing active treatment for CL. In three cases with secondarily infected ulcers, patients were treated with a 5-day course of antibiotics before sample collection.

### Lesion sampling

**Fig 1:**
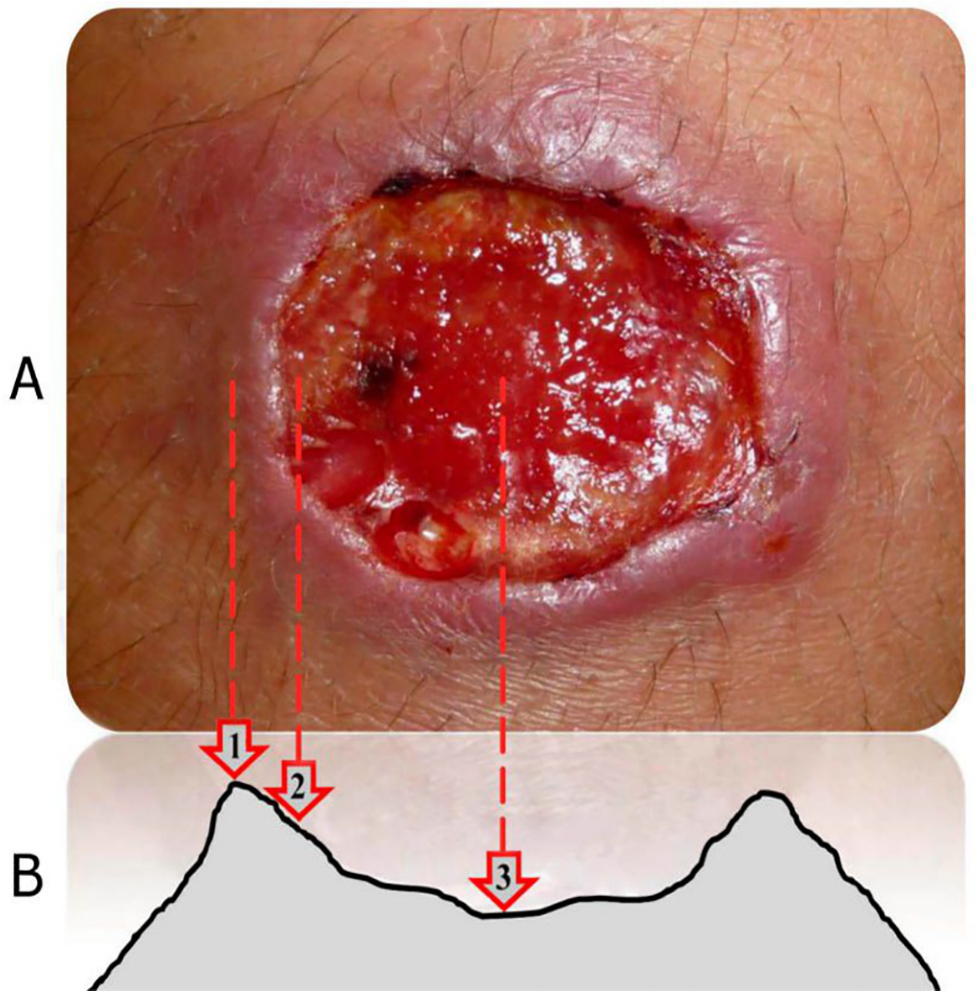
Sites of sample collection within the cutaneous ulcer. (A) Macroscopic aspect of an ulcerated lesion. (B) Schematic representation of a typical CL ulcer. The sites where samples were collected are indicated: border (1), base (2), and center (3) of the ulcer. Figure adapted from: Zvietcovich et al. [32].

In order to analyze the distribution and load of *Leishmania* amastigotes within the cutaneous lesion, samples were collected from 3 different sites, in the following order: the center of the ulcer, the base (inner border) of the ulcer, and the raised border of the ulcer (Fig 1); using a randomly chosen coordinate defined as North, South, East or West, taking as reference the lateral and longitudinal axes of the human body. If the patient had more than one lesion, the most active and typical indurated ulcer was selected. Eight specimens were collected from a single lesion per patient: a punch biopsy and a dermal scraping from each of the 3 lesion sites, and a cytology brush from each the center and base of the ulcer. The order of sampling was: biopsy, scraping, and cytology brush. All samples were taken by the same physician in order to avoid inter-individual variation.

Prior to sampling, lesions were cleansed with topical antiseptics, removed from any overlying scab or crust with saline solution and anesthetized with 1 cc of lidocaine 1%.

#### Biopsies

A small tissue fragment of 1.5 mm in diameter was obtained from the ulcer center, base and raised border, using a sterile disposable punch (Miltex), at a randomly chosen coordinate within the cutaneous lesion.

#### Scrapings

Lesion material was scraped from the ulcer center, base and from an incision made at the raised border, using a sterile lancet; this was done in the same coordinate but adjacent to the point from where biopsy samples were obtained.

#### Cytology brushes

A sterile cervical cytology brush (Cervisoft, Puritan Medical Products) was rolled clockwise at a single point of the ulcer center and base 5 times each in sequence in order to collect lesion cellular and exudative material, as described by Valencia et al. [15]; this was done in the same coordinate but adjacent to the point from where scrapings were obtained.

Clinical specimens were stored at −20°C in a 1.5 mL Eppendorf tube containing 700 μL 100% ethanol for subsequent molecular analysis.

### Isolation of DNA from biopsies, lancets and cytology brushes

Prior to DNA extraction, samples were centrifuged at 8000 g for 2 min and ethanol was discarded. Biopsied tissue was disaggregated with a sterile scalpel. Disaggregated tissue, lancets and cytology brushes were subjected to overnight lysis with Proteinase K and processed for DNA isolation using a column-based method (High Pure PCR template preparation kit, Roche), according to the manufacturer’s instructions. The isolated DNA was then quantified by fluorometry using the Quant–iT Broad Range dsDNA Assay kit (for biopsies) and the Quant-iT High Sensitivity dsDNA Assay kit (for scrapings and cytology brushes) on the Qubit fluorometer (Invitrogen). DNA samples were diluted to 5 ng/μL; those samples below this concentration were added directly into the PCR reaction.

### Parasite species identification

Parasites were typed using the heat-shock protein 70 gene (*hsp70*) PCR-N variant followed by restriction fragment length polymorphism (RFLP) analysis using the restriction enzymes *Bsa*JI and *Rsa*I as in Montalvo et al. [33].

### Detection and quantification of *Leishmania* (*Viannia*) spp.

We applied a SYBR Green-based qPCR assay targeting kDNA minicircles to detect and quantify *Leishmania* (*Viannia*) parasites in clinical samples, as previously described [26]. Each kDNA-qPCR run included a standard curve of *L*. (*V*.) *braziliensis* (MHOM/BR/75/M2904) DNA ranging from 5 × 10^4^ to 5 × 10^−3^ parasite DNA equivalents/reaction (run in duplicate); a positive control with known amount of *Leishmania* parasites, which consisted of a mix of *Leishmania* DNA and human genomic DNA in order to mimic clinical specimens (run in triplicate); a negative control (human genomic DNA from peripheral blood mononuclear cells of a healthy donor; run in triplicate); and a blank (no-template control; run in triplicate). The standard curves (inter-assay reproducibility, n = 11) showed a mean square error (MSE) of ≤0.111, correlation coefficient (*r* ^*2*^) of ≥0.998 and slopes of 3.28 (mean) ± 0.05 (standard deviation), indicating a high amplification efficiency (≥1.99) (2 would indicate 100% PCR efficiency). The positive control showed a mean of 7,640 parasites and an inter-assay coefficient of variation of 7.8% (n = 11 independent runs). All clinical samples were run in duplicate; if replicates differed by a standard deviation of >0.35 in Cq (quantification cycle) values (>0.5 cycles), they were retested.

A sample was quantified when it had a Cq value falling within the range of the standard curve. The highest dilution of template of the standard curve was defined as the lower limit of quantification (LOQ). Samples with *Leishmania* DNA levels below the LOQ could be detected; they were considered positive (qualitative detection) only if their melting curves had the same profile as those of the standards included in the same experiment. The *Leishmania* parasite load was calculated as follows: [parasite DNA equivalents per reaction/amount of tissue DNA per reaction] × 10^3^, expressed as the number of *Leishmania* parasites per μg of tissue DNA.

### Statistical analysis

Frequencies and proportions were used to describe categorical variables while median and interquartile range or mean and standard deviation were used for numeric continuous variables.

To assess whether the median parasite load in clinical specimens differed significantly according to the skin lesion site or the sampling method, analyses for paired samples using Friedman (with Dunn’s post-hoc test) and Wilcoxon signed rank tests were performed. The correlation degree between the parasite load measurements in scraping and cytology brush specimens with respect to those in biopsy specimens was calculated using the Spearman’s rank correlation test. The association between the *Leishmania* load and the parasite species was evaluated using the Mann-Whitney *U* test.

Statistical analyses were performed under a 5% significance level, using the GraphPad Prism v5.02 software.

## Results

### Study population

Demographic, epidemiological, and clinical characteristics of patients are summarized in S1 Table. Thirty-one patients with laboratory confirmed CL were enrolled: 29 (93.6%) men and 2 (6.5%) women, with median age of 34 years (range 19–75 years) and median disease duration of 2 months (range 1–3 months). Median number of lesions was 1 (range 1–10), with 21 patients (67.7%) presenting with single lesions and 9 patients (29%) presenting with multiple lesions. Bacterial superinfection was present in only 3 (9.7%) lesions. Twenty-eight patients (90.3%) had a first episode of CL and only one patient (3.2%) had a reinfection. Median duration of exposure in the risk area (i.e. stay in area of endemicity) was 3 months (range 1.5 days–75 years).

### Positivity of the kDNA-qPCR assay and diagnostic tests

The kDNA-qPCR assay detected *Leishmania* DNA in 97.6% (242/248) of the examined lesion specimens. The overall qPCR positivity per lesion-analysis taking into account the 3 lesion sites and sampling methods (96.8%; 95% CI: 74.3–100.0%) was higher than that of smear microscopy (80.6%; 95% CI: 62.5–92.6%), microculture (88.5%; 95% CI: 69.9–97.6%), and LST (72.4%; 95% CI: 52.8–87.3%), whereas it was comparable to that of the qualitative kDNA PCR (96.8%; 95% CI: 83.3–99.9%).

### Positivity of the kDNA-qPCR assay according to the ulcer site and sampling method

The performance of the qPCR for *Leishmania* DNA detection (no quantification at this stage) was assessed in the 3 lesion sites and sampling methods. In the ulcer border, *Leishmania* DNA was detected by qPCR in 100% (31/31) of the scrapings and in 90% (28/31) of the biopsies. In the ulcer base, 100% (31/31) of the biopsy specimens, 97% (30/31) of the scraping specimens, and 97% (30/31) of the cytology brush specimens tested positive for *Leishmania* DNA. In the ulcer center, *Leishmania* DNA was detected in 100% (31/31) of the examined biopsies, in 100% (31/31) of the scrapings, and in 97% (30/31) of the cytology brushes.

### Comparison of parasite loads according to the ulcer site and sampling method

**Fig 2:**
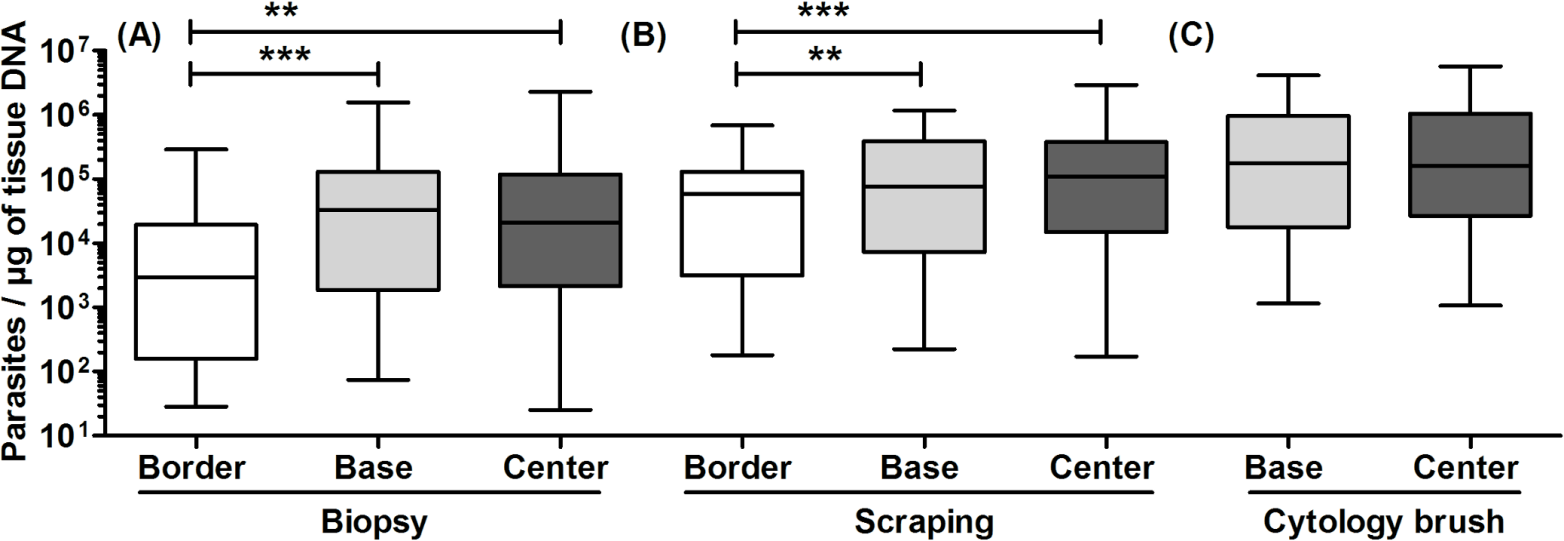
Parasite load levels in clinical samples according to skin lesion site. Data shown are the quantified paired parasite load results (8 specimens per lesion) corresponding to 28 patients. (A) Comparison of biopsy specimens taken from the ulcer border, base, and center (*P*<0.0001, Friedman test with Dunn’s post hoc test). (B) Comparison of dermal scraping specimens taken from the ulcer border, base, and center (*P* = 0.0002, Friedman test with Dunn’s post hoc test). The asterisks shown in A and B indicate statistically significant differences between corresponding groups according to Dunn’s post hoc test. (C) Comparison of cytology brush specimens taken from the ulcer base and center (*P* = 0.07, Wilcoxon signed rank test).

The kDNA-qPCR assay allowed the quantification of the parasite load in 238 out of 248 lesion specimens (96%). As for the 10 specimens that could not be quantified, 4 corresponded to dermal scrapings with detectable but not quantifiable parasite load, whereas 6 specimens among biopsies, scrapings and cytology brushes were qPCR negative. These 10 specimens corresponded to 3 patients. After exclusion of those 3 patients from the analysis, the quantified paired parasite load results (8 specimens per lesion) corresponding to 28 patients were used for parasite load assessment across ulcer sites and sampling methods. The parasite load (PL) in skin lesion specimens varied from 2.53 × 10^1^ to 5.72 × 10^6^ parasites per μg of tissue DNA. The PL levels per skin lesion site and sampling method are given in Table 1 and depicted graphically in Fig 2.

**Table 1:** *Leishmania* parasite load levels per skin lesion site and sampling method.

Skin lesion site	Sampling method	Parasite load^‡^		
		Median	IQR	Range
Raised border^¶^	Biopsy^¥^	2.96 × 10^3^	1.59 × 10^2^–1.94 × 10^4^	2.87 × 10^1–^2.88 × 10^5^
	Scraping^†^	5.96 × 10^4^	3.14 × 10^3^–1.31 × 10^5^	1.81 × 10^2–^6.84 × 10^5^
Base (inner border)*	Biopsy^¥^	3.33 × 10^4^	1.86 × 10^3–^1.32 × 10^5^	7.44 × 10^1–^1.56 × 10^6^
	Scraping^†^	7.61 × 10^4^	7.29 × 10^3–^3.90 × 10^5^	2.24 × 10^2–^1.17 × 10^6^
	Cytology brush^§^	1.76 × 10^5^	1.76 × 10^4–^9.67 × 10^5^	1.14 × 10^3–^4.12 × 10^6^
Center*	Biopsy^¥^	2.11 × 10^4^	2.14 × 10^3–^1.19 × 10^5^	2.53 × 10^1–^2.29 × 10^6^
	Scraping^†^	1.11 × 10^5^	1.50 × 10^4–^3.78 × 10^5^	1.72 × 10^2–^2.92 × 10^6^
	Cytology brush^§^	1.61 × 10^5^	2.69 × 10^4–^1.04 × 10^6^	1.07 × 10^3–^5.72 × 10^6^

**Note.** IQR, interquartile range (25^th^ percentile–75^th^ percentile).

Data shown are the quantified paired parasite load results (8 specimens per lesion) corresponding to 28 patients.

^‡^Number of parasites per μg of tissue DNA.

^¶^ *P*<0.0001, for the comparison of parasite loads between biopsy and scraping specimens (Wilcoxon signed rank test).

*P<0.0001, for the comparison of parasite loads among biopsy, scraping, and cytology brush specimens (Friedman test with Dunn’s post hoc test).

^¥^ *P*<0.0001, for the comparison of parasite loads among biopsy specimens of the ulcer border, base, and center (Friedman test with Dunn’s post hoc test).

^†^ *P* = 0.0002, for the comparison of parasite loads among scraping specimens of the ulcer border, base, and center (Friedman test with Dunn’s post hoc test).

^§^ *P* = 0.07, for the comparison of parasite loads between cytology brush specimens of the ulcer base and center (Wilcoxon signed rank test).

#### Parasite loads according to the ulcer site

Median PL differed among the 3 sites of the ulcer in biopsies and scrapings (*P*<0.0001 and *P* = 0.0002, respectively, Friedman test), with specimens from the ulcer base and center having significantly higher PL than those from the ulcer border (Dunn’s post-hoc test). There was no significant difference in PL between the ulcer base and center, either in biopsy specimens (*P* = 0.80, Wilcoxon signed rank test), scraping specimens (*P* = 0.43, Wilcoxon signed rank test), or in cytology brush specimens (*P* = 0.07, Wilcoxon signed rank test) (Table 1).

#### Parasite loads according to the sampling method

Median PL differed by sampling method: in both the ulcer base and center, (i) a higher PL could be quantified from cytology brushes and dermal scrapings as compared to biopsies; and (ii) cytology brushes showed a higher PL than scrapings (*P*<0.0001, Friedman test with Dunn’s post-hoc test). In the ulcer border, dermal scrapings contained a higher PL than biopsies (*P*<0.0001, Wilcoxon signed rank test) (Table 1). Parasite load measurements on biopsies vs. scrapings or cytology brushes were highly correlated in all lesion sites (Spearman’s rho range 0.75–0.93; *P*<0.0001, S2 Table), indicating that the parasite load trend was consistent across sampling methods.

### Comparison of parasite loads according to the infecting species

Causative species was identified in 29 of 31 (93.5%) patients having lesion specimens with sufficient amplifiable DNA: 20 patients were infected with *L*. *(V*.*) braziliensis*, 7 with *L*. *(V*.*) peruviana*, 1 with *L*. *(V*.*) guyanensis*, and 1 with *L*. *(V*.*) lainsoni*. There was no significant difference in PL according to the infecting species, taking into account in this analysis only the most well represented species (i.e. *L*. *(V*.*) braziliensis* and *L*. *(V*.*) peruviana*) (*P* = 0.4, Mann-Whitney *U* test) (Table 2).

**Table 2:** Causative *Leishmania* species and pre-treatment parasite load in tissue.

Species	Number	Parasite load^‡^		
		Median*	IQR	Range
*L*.*(V*.*) braziliensis*	20	1.47 × 10^5^	2.89 × 10^4^–3.72 × 10^5^	6.40 × 10^0^–1.17 × 10^6^
*L*.*(V*.*) guyanensis*	1	6.78 × 10^3^	—	—
*L*.*(V*.*) peruviana*	7	8.99 × 10^4^	8.79 × 10^3^–2.11 × 10^5^	2.40 × 10^2^–6.30 × 10^5^
*L*.*(V*.*) lainsoni*	1	3.04 × 10^3^	—	—
Unknown^¶^	2	2.40 × 10^1^	1.27 × 10^1^–3.53 × 10^1^	1.27 × 10^1^–3.53 × 10^1^

**Note.** IQR, interquartile range (25^th^ percentile–75^th^ percentile).

^‡^Number of parasites per μg of tissue DNA.

^¶^Species identification was not performed on these specimens because of insufficient concentration of amplifiable DNA based on kDNA PCR band thickness (as obtained in the qualitative, diagnostic PCR assay).

*P = 0.4, by Mann-Whitney *U* test for *L*. *(V*.*) braziliensis* and *L*. *(V*.*) peruviana*.

## Discussion

Here we assessed whether the *Leishmania* (*Viannia*) parasite load differs by sampling site within CL ulcers and sampling method by means of qPCR. We observed that a significantly lower amount of parasites was quantified from the ulcer border as compared to the ulcer base and center. The fact that this finding was similarly observed with lesion biopsy and scraping specimens points out its robustness. This finding called our attention because most of available studies in the literature on skin lesions caused by Neotropical *Leishmania* parasites are based on biopsies collected from the border of the ulcer, in accordance with WHO recommendations [20], as this lesion site is regarded to likely concentrate a greater amount of parasites and viable infected mononuclear phagocytes compared to the necrotic center of the ulcer [21]. Nevertheless, consistent with our findings, in a recent study that evaluated the use of swab sampling over the ulcer coupled to qPCR for diagnosis of CL, Adams et al. [16] found indications of a greater quantity of parasite DNA in the ulcerated zone of the lesion (as compared to that found in the ulcer border using aspirate samples). Furthermore, Ramírez et al. [23] reported a significant increase in the sensitivities of microscopy and conventional PCR of dermal scrapings when samples were collected from the central region of the bottom of the ulcer rather than from the margin of the lesion. This appeared to be related to a higher parasite load and easily detectable amastigotes in that area [23].

The differences in parasite load among sites within CL ulcers revealed herein may be related to the undergoing immunopathological process within the ulcer. Studies of lesion biopsies (taken, where known, from the border of the lesion) from patients infected with *L*. (*Viannia*) parasites have shown an inflammatory infiltrate in the dermis composed mainly of lymphocytes, macrophages, and plasma cells [34–38]. *Leishmania* amastigotes were seen within dermal macrophages located in the papillary dermis [35] and mid-dermis [37]. Notably, Gutierrez et al. [38] found a significant association between necrosis, relative abundance of tissue macrophages, and the presence of amastigotes in lesions of less than 6 months’ duration. The ulcerated zone of the lesion is mainly composed of dead cells, as shown by the presence of focal necrosis of the dermis as well as epidermal disruption [34–37]. In contrast, in the raised border adjacent to the ulceration, the epidermis exhibits hyperplasia and thickening [36,38].

Despite the fact that the quantified parasite loads varied widely among examined lesion samples, we found that there was no significant difference in parasite loads with respect to *L*. (*V*.) *braziliensis* and *L*. (*V*.) *peruviana* infections demonstrated in CL lesions, a finding consistent with a previous study [26]. As these 2 *Leishmania* species can lead to different clinical prognoses [39], our data lends support to a lack of association between parasite load and the degree of pathology noted in studies on human [26] and experimental murine [40] CL. Thus, differences in pathogenicity should rely on other aspects of the host-parasite interaction.

Remarkably, compared to biopsies, we found that a higher parasite load could be quantified from cytology brushes and dermal scrapings. There are two possible explanations for this finding. First, the difference in parasite loads quantified amongst sampling methods pointed out that the *Leishmania* amastigotes would not be homogeneously distributed along the skin compartments (i.e. cross-sectional), with a higher abundance of parasites to be found in the upper layers of the dermis. This is corroborated by other studies that showed that the cell types in the CL lesion infiltrates were non-randomly distributed, with macrophages and parasites being most frequently found in the mid-dermis [37] as well as in the papillary dermis [35]. Second, there are differences inherent to the sampling instrument. Skin punch biopsy is the only method that allows to sample the full-thickness skin; therefore, the ratio of human host DNA to parasite DNA in this diagnostic specimen is several-fold higher compared to scrapings and cytology brushes. This can decrease the sensitivity of detection of the pathogen in clinical samples [19,41,42]. In contrast, scraping and cytology brush sampling is more superficial and allows to recover both cellular material and tissue fluid [15]; these features reduce the proportion of host cells resulting in improved parasite DNA representation relative to the human host DNA.

Our finding that scrapings and cytology brushes outperform the invasive biopsy in terms of the parasite load quantified is particularly important when considering that invasive specimen collection is a traumatic procedure to the patient frequently associated with risks of bleeding and infection; it entails the risk of body fluid exposure to the healthcare worker via needle stick injury, and is difficult to perform in the pediatric population [13,15,43,44]. Furthermore, it is a complex medical procedure performed by trained medical personnel, normally a dermatologist, and is difficult to perform routinely in endemic settings. Cytology brushes offer the advantage over biopsies and scrapings of being non-invasive, easy to perform and well tolerated by the patient [15,44]. This makes them an attractive alternative not only for diagnosis of CL but also for monitoring patients’ response to treatment (through assessment of parasite load kinetics). Such an applicability of cytology brush sampling coupled to kDNA-qPCR has been recently evaluated in a cohort of Peruvian patients with mucosal leishmaniasis [45]. From a practical point of view, our data herein also indicate that samples for routine laboratory diagnosis or an eventual post-treatment follow-up of CL patients can be easily and safely obtained from the ulcer base and center by use of less invasive means, thus obviating the need for any skin incision from the lesion border.

The qPCR assay employed herein targets a multicopy conserved region of minicircle kDNA common to *Leishmania* (*Viannia*) species, present at about 10,000 copies per amastigote [46], thereby allowing to quantify the number of parasites present in the ulcer with high sensitivity. The *Leishmania* kDNA levels detected and quantified in the lesion specimens are likely indicative of the presence of viable parasites, since nuclear and kinetoplast *Leishmania* DNA are rapidly degraded following amastigote death [47]. The variability of the number of kDNA minicircle targets [26,48] was not assessed in the lesion specimens examined here. Nonetheless, our data analysis took into account paired samples (those taken from a same lesion of a patient), which allowed a more accurate estimation of parasite load levels in the ulcer across subjects.

In this study, only patients with recent onset CL (≤3 months) were enrolled. That early stage of CL is associated with a positive parasitological diagnosis and a high parasite load; conversely, chronic CL (>6 months) is characterized by a scarcity of parasites in lesions [10,23,26,38]. Thus, further studies covering both acute and chronic stages of CL caused by *L*. (*Viannia*) parasites are needed to confirm and expand our results. Nevertheless, this study is valuable as it is, to our knowledge, the first report that assesses quantitatively whether the *Leishmania* parasite load differs by both site of sample collection within the skin ulcer and sampling method by means of qPCR.

Our results herein are applicable to ulcerative skin lesions, which represent the most frequent form of localized CL in Latin America. In areas where leishmaniasis is endemic, a smaller number of patients present with other types of cutaneous manifestations (nodular, verrucous, plaques, and papular lesions), either as primarily presentation or in addition to the ulcerative lesion [13,49,50]. Future studies assessing the parasite load in these other types of lesions covering different stages of the disease will add to our understanding of a polymorphic skin disease as CL is.

Our data reveal a picture of the CL ulcer being a complex place, where the process of survival of *Leishmania* amastigotes is occurring, with abundant amastigotes in a highly necrotic tissue. Future studies based on morphometric analysis of histopathological sections are needed to establish the in situ location and quantity of parasites in relation to cellular infiltrates in the ulcerated zone of the lesion, and during different stages of the disease. This may further our understanding of the dynamics of infection in human CL due to *L*. (*Viannia*) species.

## Supporting Information

S1 Table Demographic, epidemiological and clinical characteristics of the 31 enrolled patients with CL. Note. Data are number (%) of cases, unless otherwise indicated. *n*, no. of patients; SD, standard deviation; IQR, interquartile range (25^th^ percentile–75^th^ percentile).
(DOCX)

S2 Table Correlation between parasite load measurements on biopsy specimens vs. scraping or cytology brush specimens. Note. CI, confidence interval.
(DOCX)
