# Supplementary material for: Quantification of Leishmania (Viannia) Kinetoplast DNA in Ulcers of Cutaneous Leishmaniasis Reveals Inter-site and Inter-sampling Variability in Parasite Load
Source: PLoS Negl Trop Dis. 2015 Jul 23;9(7):e0003936. doi: 10.1371/journal.pntd.0003936 (PMC4512720; doi:10.1371/journal.pntd.0003936)
Supplement: S1 Table — Note. Data are number (%) of cases, unless otherwise indicated. n, no. of patients; SD, standard deviation; IQR, interquartile range (25th percentile–75th percentile). (DOCX) [file pntd.0003936.s001.docx]

**S1 Table.** Demographic, epidemiological and clinical characteristics of the 31 enrolled patients with CL.

| **Characteristic** | **Value** |
| --- | --- |
| Age (years); *n*=29 |  |
| Mean ± SD | 38.5 ± 16.5 |
| Median (IQR) | 34 (25–48) |
| Range | 19–75 |
| Sex |  |
| Men | 29 (93.6%) |
| Women | 2 (6.5%) |
| Region of disease acquisition |  |
| Central and northern Coast (Ancash, Lima, Piura/Lambayeque) | 6 (19.4%) |
| Southern Andes (Apurimac, Cusco) | 5 (16.1%) |
| North Jungle (Loreto, San Martin) | 11 (35.5%) |
| Central Jungle (Ucayali) | 2 (6.5%) |
| South Jungle (Madre de Dios) | 5 (16.1%) |
| Unknown | 2 (6.5%) |
| Duration of exposure in the risk area (days); *n*=28 |  |
| Mean ± SD | 3366 ± 7509.1 |
| Median (IQR) | 90 (52.5–225) |
| Range | 1.5 days–75 years |
| Duration of disease (months); *n*=29 |  |
| Mean ± SD | 2.2 ± 0.7 |
| Median (IQR) | 2 (1.5–3) |
| Range | 1–3 |
| Number of lesions; *n*=30 |  |
| Mean ± SD | 1.9 ± 2 |
| Median (IQR) | 1 (1–2) |
| Range | 1–10 |
| Single | 21 (67.7%) |
| Multiple | 9 (29%) |
| Lesion size (cm); *n*=27 |  |
| Mean ± SD | 4.2 ± 2.8 |
| Median (IQR) | 3.4 (2.5–4.6) |
| Range | 1–12.5 |
| Location of lesion |  |
| Face | 1 (3.2%) |
| Upper extremities | 11 (35.5%) |
| Lower extremities | 16 (51.6%) |
| Trunk | 2 (6.5%) |
| Unknown | 1 (3.2%) |
| Regional lymph nodes |  |
| Yes | 13 (41.9%) |
| No | 15 (48.4%) |
| Unknown | 3 (9.7%) |
| Bacterial/fungal superinfection |  |
| Yes | 3 (9.7%) |
| No | 26 (83.9%) |
| Unknown | 2 (6.5%) |

**Note.** Data are number (%) of cases, unless otherwise indicated. *n*, no. of patients; SD, standard deviation;

IQR, interquartile range (25^th^ percentile–75^th^ percentile).
