# Supplementary material for: Quantification of Leishmania (Viannia) Kinetoplast DNA in Ulcers of Cutaneous Leishmaniasis Reveals Inter-site and Inter-sampling Variability in Parasite Load
Source: PLoS Negl Trop Dis. 2015 Jul 23;9(7):e0003936. doi: 10.1371/journal.pntd.0003936 (PMC4512720; doi:10.1371/journal.pntd.0003936)
Supplement: S2 Table — Note. CI, confidence interval. (DOCX) [file pntd.0003936.s002.docx]

**S2 Table.** Correlation between parasite load measurements on biopsy specimens vs. scraping or cytology brush specimens.

| **Skin lesion site** | **Sampling method** | **Spearman's rho** | **95% CI**  **of Spearman’s rho** | ***P*-value** |
| --- | --- | --- | --- | --- |
| Raised border | Scraping *vs.* Biopsy | 0.75 | 51.64 – 88.07 | < 0.0001 |
| Base (inner border) | Scraping *vs.* Biopsy | 0.87 | 72.98 – 93.97 | < 0.0001 |
|  | Cytology brush *vs*. Biopsy | 0.93 | 85.62 – 96.97 | < 0.0001 |
| Center | Scraping *vs.* Biopsy | 0.88 | 74.25 – 94.29 | < 0.0001 |
|  | Cytology brush *vs.* Biopsy | 0.87 | 72.35 – 93.81 | < 0.0001 |

**Note.** CI, confidence interval.
